# Molecular detection and characterization of filarioid nematodes in wild armadillos and anteaters from Central-Western Brazil

**DOI:** 10.1007/s11259-026-11570-0

**Published:** 2026-10-07

**Authors:** Jovêncio Mateus Sada, Danilo Kluyber, João Vitor dos Santos Alves da Silva, Ana Claúdia Calchi, Daniel Antônio Braga Lee, Mayara Caiaffa, Grazielle Soresini, Mario H. Alves, Ana Carolina Monteirinho Lobo, Arnaud Desbiez, Expedito José de Albuquerque Luna, Olivier Duron, Rosangela Zacarias Machado, Júlio A. Benavides, Marcos Rogério André

**Affiliations:** 1 Vector-Borne Bioagents Laboratory (VBBL), Department of Pathology, Reproduction and One Health, School of Agricultural and Veterinarian Sciences (FCAV), São Paulo State University (UNESP), Jaboticabal Campus, Via Prof. Access Paulo Donato Castellane, s/n, Rural Zone, Jaboticabal, SP 14884-900 Brazil; 2 Instituto de Conservação de Animais Silvestres – ICAS, Campo Grande, Brazil; 3 Naples Zoo, Naples, Florida USA; 4 School of Medicine, Institute of Tropical Medicine, University of São Paulo, São Paulo, SP Brazil; 5 UMR MIVEGEC, University of Montpellier, IRD, CNRS, 911 Av. Agropolis CP34394, Montpellier, France; 6 Departamento de Biologia Animal, UNICAMP, Campinas, SP Brazil; 7 Department of Veterinary Medicine, Università degli Studi di Bari Aldo Moro, Valenzano, Italy; 8 Department of Veterinary Surgery and Animal Reproduction, School of Veterinary Medicine and Animal Science, São Paulo State University- UNESP, Botucatu, São Paulo 18618-81 Brazil; 9 One Health Institute and Doctorado en Medicina de la Conservación, Faculty of Life Sciences, Universidad Andrés Bello, República 440, Piso 1, Santiago, Región Metropolitana 8370251 Chile

**Keywords:** Nematodes, Molecular diagnosis, Pilosa, Cingulata, Wildlife

## Abstract

Armadillos and anteaters usually interact with a wide range of ectoparasites and associated pathogens. Combined with their unique physiological and ecological characteristics, these interactions make them potential hosts for a diverse range of vector-borne agents (VBA). Despite this, relatively few studies have investigated the occurrence of VBA in mammals of the superorder Xenarthra in Brazil. The present study aimed to investigate the occurrence and phylogenetic assessment of filarioid nematodes in wild armadillos and anteaters sampled in the state of Mato Grosso do Sul, Central-Western Brazil. A total of 123 biological samples, comprising 105 blood and 18 spleen samples, were analyzed. The spleen samples were collected from five southern tamanduas (*Tamandua tetradactyla*) and 13 giant anteaters (*Myrmecophaga tridactyla*), whereas the blood samples were obtained from 42 six-banded armadillos (*Euphractus sexcinctus*), 15 giant armadillos (*Priodontes maximus*), and 44 giant anteaters (*M. tridactyla*). Following DNA extraction and confirmation of DNA quality by PCR amplification of the mammalian *gapdh* gene, all samples were screened for filarioid DNA using a PCR assay targeting the mitochondrial *cox1* (cytochrome c oxidase subunit I) gene. Samples testing positive in the screening assay were subsequently subjected to PCR amplification of the mitochondrial 12 S rRNA gene for further molecular characterization. Overall, 53/123 (43.08%) samples were positive in the *cox1*-based screening PCR, including 24/42 *E. sexcinctus*, 4/19 *P. maximus*, 23/57 *M. tridactyla*, and 2/5 *T. tetradactyla* samples. Of these, 14/53 (26.41%) were also positive in the PCR targeting the filiarioid 12 S rRNA gene, including 11 *E. sexcinctus*, one *P. maximus*, one *M. tridactyla*, and one *T. tetradactyla* sample. Phylogenetic analysis based on the *cox1* gene showed that the five sequences obtained from *E. sexcinctus* and the single sequence from *P. maximus* clustered together in a distinct clade closely related to *Onchocerca* spp., whereas the sequence obtained from *T. tetradactyla* formed a separate, isolated lineage. In contrast, phylogenetic analysis based on the 12 S rRNA gene positioned the sequences detected in *E. sexcinctus* and *P. maximus* within the same clade as a unclassified Filariidae previously detected in *M. tridactyla* from Mato Grosso do Sul. To the best of authors’ knowledge, this is the first molecular detection of filarioid nematodes in armadillos. Furthermore, phylogenetic analyses based on two independent molecular markers (*cox1* and 12 S rRNA) revealed the occurrence of distinct filarioid lineages associated with mammals of the superorder Xenarthra in Brazil, expanding current knowledge on the diversity and host associations of these parasites in neotropical wildlife.

## Introduction

Filarioid nematodes (Spirurida: Filariidae/Onchocercidae) are vector-borne parasites transmitted to vertebrate hosts by hematophagous arthropods, including mosquitoes, black flies, biting midges, and ticks (Otranto et al. 2012; Brianti et al. 2022; Bezerra-Santos et al. 2022; Binetruy and Duron 2023). These parasites are of considerable veterinary and public health importance due to their ability to infect a broad range of domestic animals, wildlife, and humans (Pupić-Bakrač et al. 2021; Soares et al. 2022).

Despite Brazil harboring one of the world’s richest wildlife biodiversity, studies investigating the diversity of filarioid nematodes in wild mammals remain scarce. To date, *Mansonella* spp. and *Brugia* spp. have been molecularly detected in coatis (*Nasua nasua*) (Moraes et al. 2022; Perles et al. 2023). Filarioids phylogenetically related to *Dipetalonema graciliformis* have been identified in non-human primates, including the brown howler monkey (*Alouatta guariba clamitans*) and the black-horned capuchin (*Sapajus nigritus*) (Ehlers et al. 2023). Distinct filarioid haplotypes closely related to *Dirofilaria immitis*, *Breinlia*, and *Malayfilaria* have been reported in wild felids (Fagundes-Moreira et al. 2024; Santana et al. 2025). Likewise, filarioids phylogenetically associated with *Dirofilaria* and *Onchocerca* have been detected in wild canids (Santana et al. 2025), whereas *Litomosoides* spp. has been molecularly identified in non-hematophagous bats (Costa et al. 2022). Similarly, in neighboring French Guiana, several tick-borne filarioids circulating in wildlife have also been detected, further supporting the diversity of sylvatic filarial nematodes in South America (Binetruy and Duron 2023; Bruley and Duron 2024).

Although several arthropod-borne pathogens, including piroplasmids, *Anaplasma* spp., *Ehrlichia* spp., *Bartonella* spp., *Borrelia* spp., and hemotropic mycoplasmas (hemoplasmas), have been previously detected in armadillos and anteaters in Brazil (Calchi et al. 2020a; 2020b, 2023; de Oliveira et al. 2022; Buysse et al. 2024; Sada et al. 2024; Calchi et al. 2025), information regarding filarioid nematodes in these hosts remains extremely limited. To date, only one recent study reported the molecular detection of filarioid DNA in giant anteaters (*Myrmecophaga tridactyla*) from Central-Western Brazil (Santana et al. 2025). Therefore, the present study aimed to investigate the occurrence and phylogenetic assessment of filarioid nematodes in wild armadillos and anteaters sampled in the state of Mato Grosso do Sul, Central-Western Brazil.

## Materials and methods

### Sampled species and study area

Between 2017 and 2024, a total of 123 biological samples (105 blood and 18 spleen samples) were collected from mammals of the superorder Xenarthra (orders Cingulata and Pilosa) in the Nhecolândia subregion of the Pantanal and in the Cerrado biomes, Mato Grosso do Sul State, Central-Western Brazil. Sample collection was conducted as part of the Programa de Conservação do Tatu-canastra (PCTC) and the Projeto Bandeiras e Rodovias, coordinated by the Instituto de Conservação de Animais Silvestres (ICAS).

**Fig. 1:**
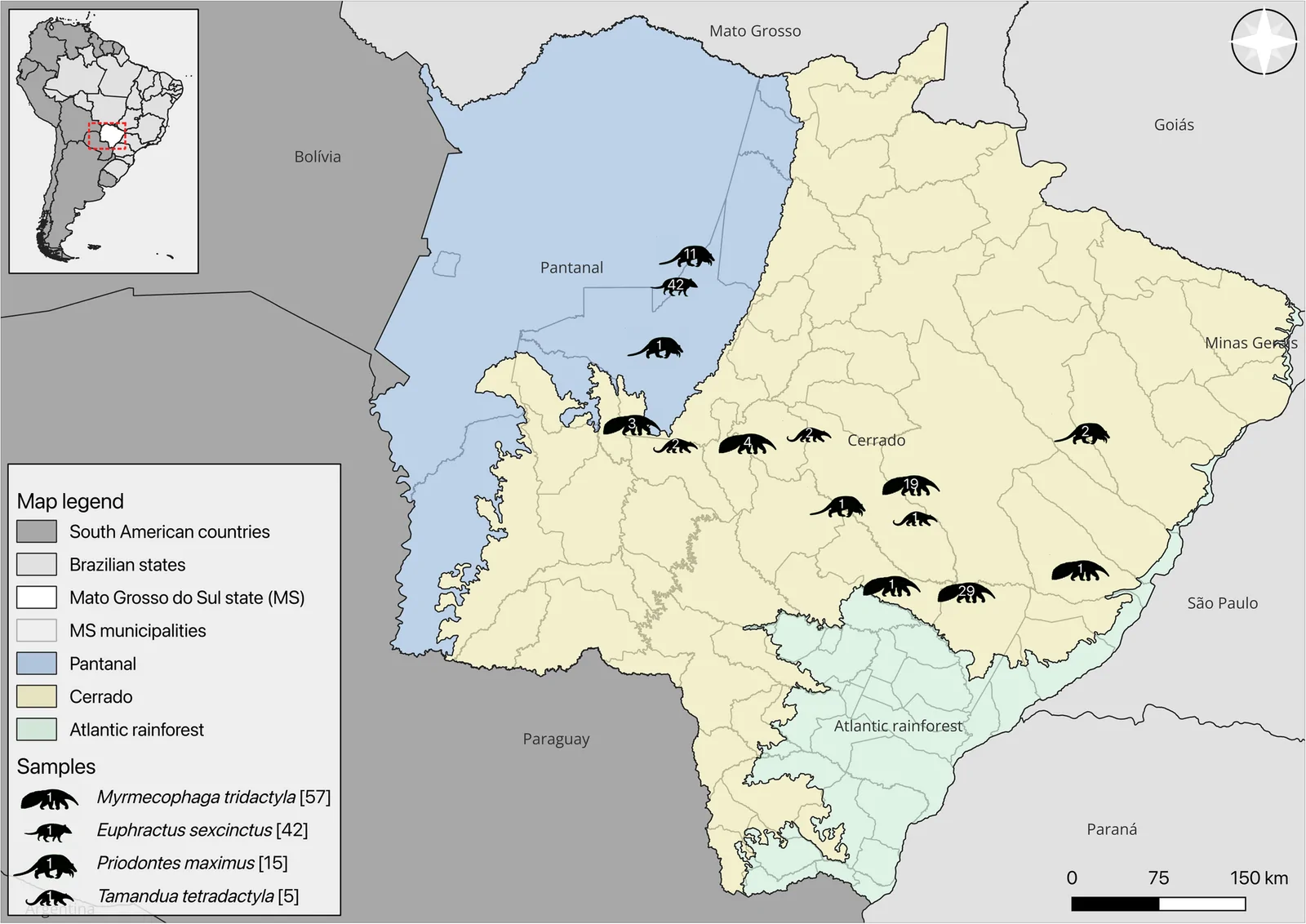
Geographic distribution of the sampling sites in the Nhecolândia subregion, encompassing the Pantanal and Cerrado biomes, Mato Grosso do Sul State, Brazil, where blood and spleen samples from Xenarthra were collected between 2017 and 2024. Sampling locations are identified according to the municipalities included in the study, and the number of individuals sampled at each locality is indicated for *Euphractus sexcinctus*, *Priodontes maximus*, *Myrmecophaga tridactyla*, and *Tamandua tetradactyla*. The inset map shows the location of Mato Grosso do Sul State within Brazil, as well as the distribution of the Pantanal and Cerrado biomes. Symbols represent the sampling sites included in the present study and correspond to the geographic origin of the animals from which biological samples were obtained. The map was created using QGIS software (version 4.1-Master development build)

The spleen samples were obtained during necropsies of five southern tamanduas (*Tamandua tetradactyla*) and 13 giant anteaters (*Myrmecophaga tridactyla*) that had died as a result of vehicle collisions on highways. In addition, blood samples were collected from alive 42 six-banded armadillos (*Euphractus sexcinctus*), 15 giant armadillos (*Priodontes maximus*), and 44 giant anteaters (*M. tridactyla*) that were live-captured, sampled, and subsequently released at their capture sites according to previously published protocols (Kluyber et al. 2020; Desbiez et al. 2021) (Fig. 1). Spleen tissue fragments and blood samples were stored in RNase- and DNase-free KASVI^®^ microtubes at − 70 °C until DNA extraction.

### DNA extraction

DNA was extracted from 10 mg of each spleen tissue and 200 µL of each blood sample using the DNeasy Blood & Tissue Kit (Qiagen, Valencia, CA, USA), according to the manufacturer’s instructions. DNA concentration and purity were assessed using a NanoDrop spectrophotometer (Thermo Fisher Scientific, Waltham, MA, USA). In order to verify the presence of amplifiable DNA and to discard the presence of PCR inhibitors, DNA samples were subjected to a conventional PCR (cPCR) assay targeting the mammalian endogenous glyceraldehyde-3-phosphate dehydrogenase (*gapdh*) gene (Birkenheuer et al. 2003).

### PCR assays for the molecular detection and characterization of filarioid nematodes

Samples positive for the mammalian *gapdh* gene were screened for filarioid DNA using a conventional PCR assay. The assay targeted a 689-bp fragment of the mitochondrial *cox1* (cytochrome c oxidase subunit I) gene, following the protocol described by Casiraghi et al. (2001). Each 25-µL reaction contained 5 µL of template DNA, 1.25 U of Taq DNA polymerase (Invitrogen^®^, Carlsbad, CA, USA), 1× PCR buffer (100 mM Tris-HCl, pH 9.0, 500 mM KCl), 0.2 mM of each deoxynucleotide triphosphate (dATP, dTTP, dCTP, and dGTP), 1.5 mM MgCl₂, 0.5 µM of each primer, and sterile ultrapure water to the final reaction volume. Samples yielding positive results in the *cox1*-based screening assay were subsequently subjected to a conventional PCR targeting a 450-bp fragment of the mitochondrial 12 S rRNA gene, as previously described by Casiraghi et al. (2004), for further molecular characterization. DNA from *Dirofilaria immitis*, kindly provided by Prof. Dr. Norma Labarthe (FIOCRUZ, Rio de Janeiro, Brazil), was used as the positive control. Sterile ultrapure water was included as a negative control in all PCR assays.

PCR amplicons were separated by horizontal electrophoresis on 1.0% agarose gels stained with ethidium bromide (0.5 µg/mL) in Tris-borate-EDTA (TBE; pH 8.0) running buffer. Electrophoresis was performed at 90 V and 150 mA for 60 min. A 100-bp DNA ladder (Life Technologies^®^, São Paulo, SP, Brazil) was used as the molecular size marker. Amplified products were visualized under ultraviolet light using a ChemiDoc™ Imaging System (Bio-Rad^®^, Hercules, CA, USA) and analyzed with the corresponding image analysis software.

### Sequencing

PCR amplicons were purified using the ExoSAP-IT™ PCR Product Cleanup Reagent (Thermo Fisher Scientific, San Jose, CA, USA), according to the manufacturer’s instructions. Purified products were sequenced using the dideoxy chain-termination method (Sanger et al. 1977) on an ABI PRISM 3700 DNA Analyzer (Applied Biosystems^®^, Foster City, CA, USA) at the Center for Biological Resources and Genomic Biology (CREBIO), School of Agricultural and Veterinary Sciences (FCAV), São Paulo State University (UNESP), Jaboticabal, Brazil.

### Phylogenetic analyses

Forward and reverse sequence reads generated from each sample were assembled into consensus sequences using Phred–Phrap version 23 (Ewing and Green 1998; Ewing et al. 1998). Only nucleotide positions with a minimum Phred quality score of 20 were considered for consensus sequence construction. The resulting consensus sequences were compared with homologous sequences available in the GenBank database using the BLASTn algorithm (Altschul et al. 1990).

Consensus sequences were exported in FASTA format and aligned with homologous sequences retrieved from GenBank using MAFFT version 7 (Katoh and Standley 2013). The alignments were manually inspected and trimmed using BioEdit version 7.2 (Hall 1999). Sequence alignments were subsequently converted into NEXUS, PHYLIP, and MEGA formats using the Alignment Transformation Environment (ALTER) (Glez-Peña et al. 2010).

Phylogenetic analyses were performed using the Maximum Likelihood (ML) method implemented in IQ-TREE version 3.0.1. The best-fit nucleotide substitution model was selected using jModelTest 2 (Darriba et al. 2012). Branch support was assessed by 1,000 bootstrap replicates (Felsenstein 1985). Phylogenetic trees were edited, visualized, and rooted using an appropriate outgroup in Interactive Tree Of Life (iTOL) version 6 (Letunic and Bork 2024).

## Results

All 123 DNA samples extracted from blood and spleen tissues of armadillos and anteaters tested positive in the PCR assay targeting the endogenous mammalian *gapdh* gene, confirming the presence of amplifiable DNA. Of these, 53/123 (43.08%) samples were positive in the filarioid screening PCR targeting the mitochondrial *cox1* gene, including 24/42 blood samples from *E. sexcinctus*, 4/19 blood samples from *P. maximus*, 23/57 samples from *M. tridactyla* (18 blood and five spleen samples), and 2/5 spleen samples from *T. tetradactyla*.

Among the 53 *cox1*-positive samples, 14 (26.4%) were successfully amplified in the PCR assay targeting the 12 S rRNA gene, comprising 11 DNA blood samples from *E. sexcinctus*, one DNA blood sample from *P. maximus*, one DNA spleen sample from *M. tridactyla*, and one DNA spleen sample from *T. tetradactyla*.

The results of the BLASTn analysis for the *cox1* and 12 S rRNA gene sequences are summarized in Table 1.

**Table 1:** BLASTn results of partial *cox1* and 12 S rRNA gene sequences of filarioid nematodes detected in biological samples from wild anteaters and armadillos in Central-Western Brazil

ID/ Biological sample (Genbank accession number)	Xenarthra species/State	Gene	Size (bp)	Query cover	E- value	Identity	Host	Result BLASTn	Location/Genbank accessionnumber
ES-12/ Blood (PZ782045)	*Euphractus sexcinctus/*MS	*cox-1*	656	94%	0.0	92.72%	*Cervus elaphus*	*Onchocerca skrjabini*	Switzerland/ ON854653.1
ES_34/ Blood (PZ782046)	*Euphractus sexcinctus/*MS	*cox-1*	653	95%	0.0	92.57%	*Cervus elaphus*	*Onchocerca skrjabini*	Switzerland/ ON854653.1
ES_43/ Blood (PZ782048)	*Euphractus sexcinctus/*MS	*cox-1*	645	94%	0.0	92.78%	*Cervus elaphus*	*Onchocerca skrjabini*	Switzerland/ ON854653.1
ES_48/ Blood (PZ782047)	*Euphractus sexcinctus/*MS	*cox-1*	654	95%	0.0	92.57%	*Cervus elaphus*	*Onchocerca skrjabini*	Switzerland/ ON854653.1
NEC93/ Spleen (PZ782043)	*Tamandua tetradactyla/*MS	*cox-1*	576	100%	0.0	90.62%	Macaque	*Onchocercidae* sp.	Indonesia PX995899.1
NEC99/ Spleen (PZ782044)	*Tamandua tetradactyla/*MS	*cox-1*	564	98%	0.0	91.01%	Macaque	*Onchocercidae* sp.	Indonesia PX995899.1
ES-12/ Blood (PZ694556)	*Euphractus sexcinctus/*MS	12 S rRNA	486	100%	0.0	92.02%	Cattle	*Onchocerca gibsoni*	Australia/ AY462913.1
ES-13/ Blood (PZ694553)	*Euphractus sexcinctus/*MS	12 S rRNA	486	100%	0.0	92.02%	Cattle	*Onchocerca gibsoni*	Australia/ AY462913.1
ES-31/ Blood (PZ694554)	*Euphractus sexcinctus/*MS	12 S rRNA	485	100%	0.0	92.01%	Cattle	*Onchocerca gibsoni*	Australia/ AY462913.1
ES-37/ Blood (PZ694558)	*Euphractus sexcinctus/*MS	12 S rRNA	434	99%	1e-171	92.61%	Cattle	*Onchocerca gibsoni*	Australia/ AY462913.1
ES-48/ Blood (PZ694557)	*Euphractus sexcinctus/*MS	12 S rRNA	459	99%	9e-179	92.14%	Cattle	*Onchocerca gibsoni*	Australia/ AY462913.1
TC38/ Blood (PZ694555)	*Priodontes maximus/*MS	12 S rRNA	484	99%	0.0	91.%	Cattle	*Onchocerca gibsoni*	Australia/ AY462913.1

*MS Mato Grosso do Sul, *ID* identification

**Fig. 2:**
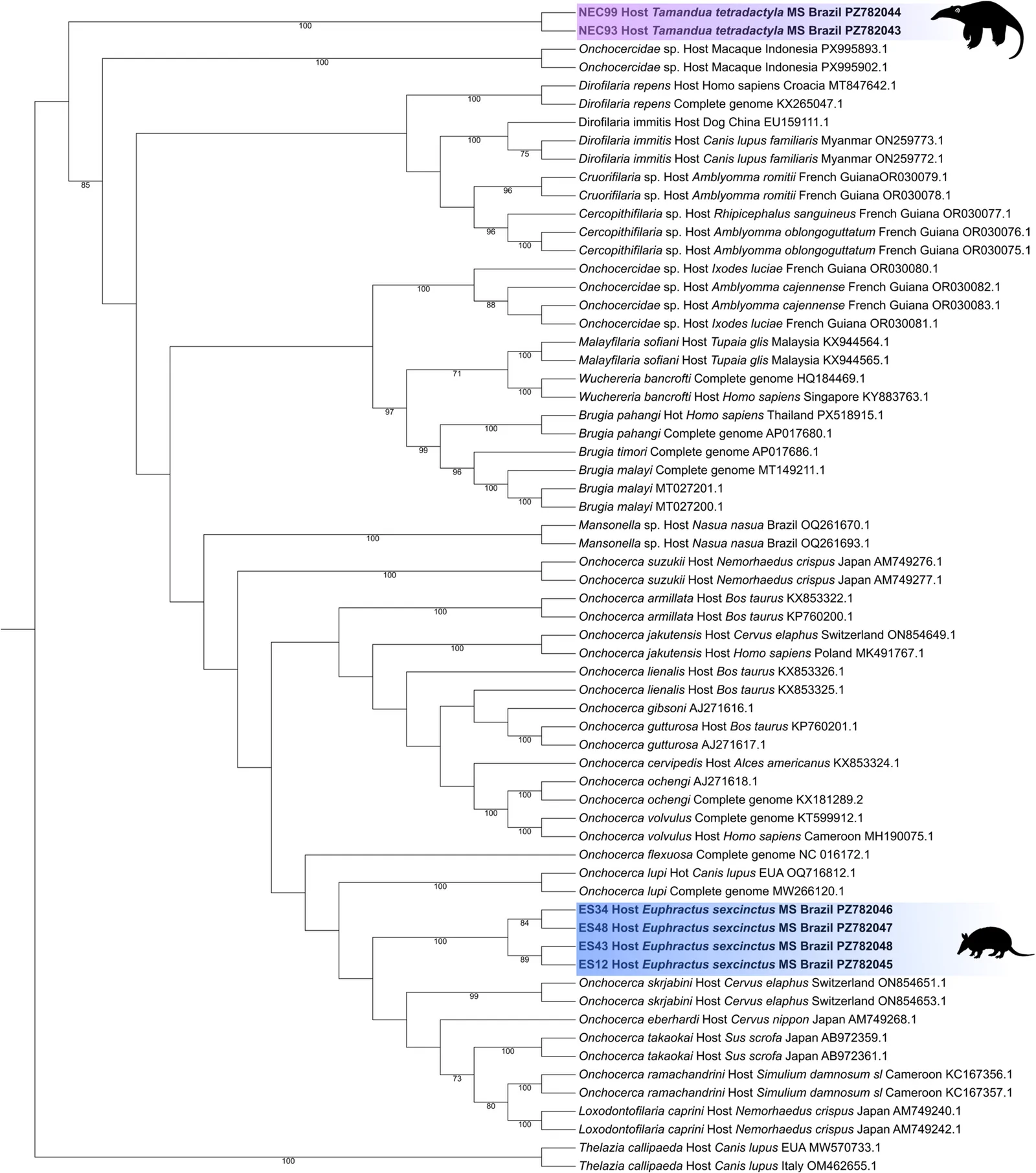
Maximum likelihood phylogenetic tree inferred from partial mitochondrial *cox1* gene sequences of filarioid nematodes detected in *Tamandua tetradactyla*, and *Euphractus sexcinctus* in Mato Grosso do Sul State, Brazil, together with representative filarioid sequences retrieved from GenBank. Phylogenetic reconstruction was performed using the GTR + F+I+G4 nucleotide substitution model with 1,000 bootstrap replicates. Bootstrap support values are shown at the nodes, with only values ≥ 70% displayed. Sequences generated in the present study are highlighted in rose (anteaters) and blue (armadillos) and are identified by their respective sample codes, host species, sampling locality and Genbank Accession Numbers. *Thelazia callipaeda* sequences were used as the outgroup to root the tree. The scale bar indicates the number of nucleotide substitutions per site

In the Maximum Likelihood phylogenetic analysis using the GTR + F+I+G4 evolutionary model, based on a 676-bp alignment of the mitochondrial *cox1* gene, the filarioid sequences detected in blood samples from the six-banded armadillo (*E. sexcinctus*) formed a well-supported monophyletic clade (bootstrap value = 100) closely related to sequences of the genus *Onchocerca*. In contrast, sequences obtained from *T. tetradactyla* formed a distinct and isolated clade (Fig. 2).

**Fig. 3:**
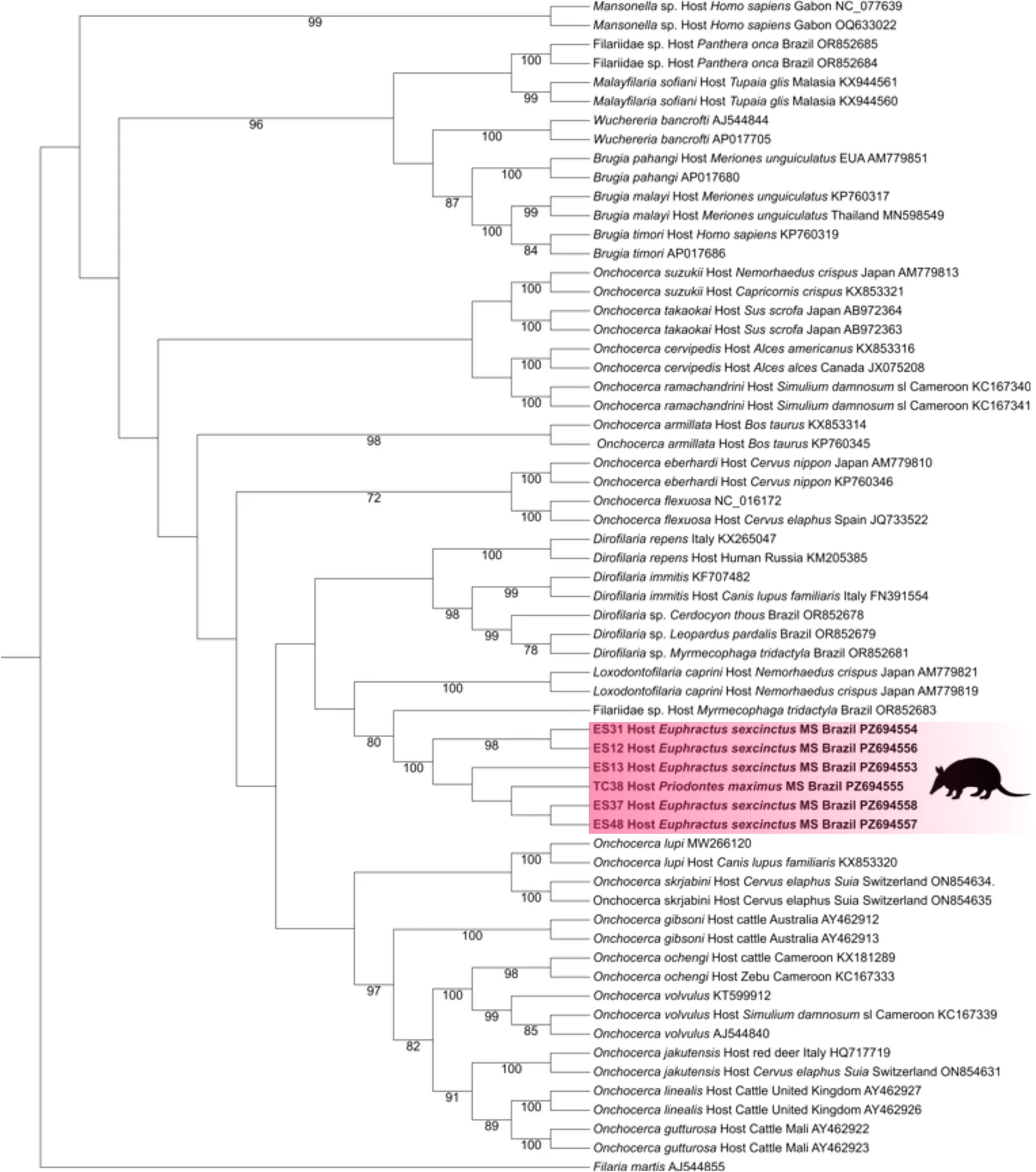
Maximum likelihood phylogenetic tree inferred from partial mitochondrial 12 S rRNA gene sequences of filarioid nematodes detected in *Euphractus sexcinctus* and *Priodontes maximus* in Mato Grosso do Sul State, Brazil, together with representative filarioid sequences retrieved from GenBank. Phylogenetic reconstruction was performed using the GTR + I+G nucleotide substitution model with 1,000 bootstrap replicates. Bootstrap support values are shown at the nodes, with only values ≥ 70% displayed. Sequences generated in the present study are highlighted in rose (armadillos) and are identified by their respective sample codes, host species, sampling locality and Genbank Accession Numbers. A *Filaria martis* sequence was used as the outgroup to root the tree. The scale bar represents the estimated number of nucleotide substitutions per site

In the Maximum Likelihood phylogenetic analysis under the GTR + I+G evolutionary model, based on a 527-bp alignment of the mitochondrial 12 S rRNA gene, the filarioid sequences detected in blood samples from the six-banded armadillos and the giant armadillos clustered together in a sister clade with high bootstrap support, grouping with an unclassified Filariidae sequence previously detected in a giant anteater (*M. tridactyla*) (OR852683) from Central-Western Brazil (Fig. 3).

## Discussion

The results of the present study revealed a high occurrence of filarioids in armadillos (49.12%) and anteaters (40.32%) from Mato Grosso do Sul State, Central-Western Brazil, suggesting that these infections may be endemic in the region and that these mammals play an important role in maintaining filarioid parasites in natural ecosystems. Recent studies have demonstrated that filarioids are widely distributed among diverse groups of Brazilian wild mammals, including coatis, wild canids, felids, non-human primates, and bats, highlighting the ecological importance of these hosts in the epidemiology and maintenance of filarial infections (Moraes et al. 2017, 2022; Costa et al. 2022; Perles et al. 2023; Fagundes-Moreira et al. 2024; Santana et al. 2025).

Although 53 samples tested positive for the *cox1* gene, only 14 of them were successfully amplified for the 12 S rRNA gene. This discrepancy indicates that the employed 12 S rRNA-based primers amplified only a subset of the filarioid diversity present in the examined armadillos and anteaters. Given that filarioids infecting Xenarthra remain poorly characterized at the molecular level, it is plausible that the high genetic diversity of these lineages reduced primer annealing efficiency, thereby limiting amplification of this marker. A similar pattern was reported by Santana et al. (2025), who investigated filarioids infecting multiple wild mammal species across different Brazilian biomes and observed differences in amplification success between mitochondrial markers, with several samples yielding positive results for one marker but not for another. These findings reinforce the importance of employing multiple molecular markers when investigating the diversity and phylogenetic relationships of filarioids.

Both the BLASTn (92% nucleotide identity with *Onchocerca skrjabini*) and the phylogenetic analyses based on partial *cox1* sequences indicate that the filarioids detected in armadillos belong to a lineage closely related to the genus *Onchocerca*. In contrast, the 12 S rRNA sequences showed their highest nucleotide identity (> 91%) with *Onchocerca gibsoni* and clustered in a distinct clade closely related to an unclassified Filariidae. previously detected in a giant anteater (*M. tridactyla*) from Central-Western Brazil. This discrepancy between markers is not unexpected, as *cox1* evolves more rapidly and generally provides higher discriminatory power for species delimitation, whereas 12 S rRNA is more conserved and typically offers lower resolution at the species level. Furthermore, the relatively low nucleotide identities observed for both markers (approximately 91–92%) suggest that the filarioids detected in this study may represent previously uncharacterized evolutionary lineages. Nevertheless, the formal description of novel species within the family Onchocercidae requires an integrative taxonomic approach combining morphological characterization, phylogenetic assessment based on multiple molecular markers, and broader specimens sampling.

Given the pathogenic and zoonotic potential of *Onchocerca* species, the filarioids detected in armadillos warrant further investigation. In Brazil, *Onchocerca volvulus*, the etiological agent of human onchocerciasis transmitted by black flies (*Simulium* spp.), is responsible for ocular alterations that may progress to blindness, as well as cutaneous lesions and lymphatic disorders in humans with transmission restricted to the Yanomami Indigenous Territory, in the far north of the country, on the border with Venezuela (Herzog-Neto et al. 2014; Viana et al. 2023; Araujo et al. 2024). *Onchocerca lupi*, in turn, has been associated with ocular onchocerciasis in dogs and, occasionally, in humans (Ilhan et al. 2013; Roe et al. 2023).

Interestingly, the *cox1* sequences obtained from the southern tamandua (*T. tetradactyla*) formed a well-supported, independent clade, suggesting the occurrence of previously undescribed filarioids infecting anteaters in Brazil. Although filarioids have recently been molecularly reported in *M. tridactyla* (Santana et al. 2025), the absence of corresponding *cox1* sequences in GenBank precluded direct comparisons with the lineage detected in *T. tetradactyla*. When compared with filarioid (*Cercopithifilaria*, *Cruorifilaria*, and *Dipetalonema*-like filarioids) *cox1* sequences detected in ticks from French Guiana (Binetruy and Duron 2023), the sequences obtained herein did not cluster with any of these taxa; instead, they formed a distinct lineage, further supporting the hypothesis that they represent undescribed filarioid lineages.

These findings highlight the limited representation of Xenarthra-associated filarioid sequences in public databases and underscore the need for further studies using multiple molecular markers to better characterize the diversity and resolve the evolutionary relationships of filarioids infecting armadillos and anteaters from Brazil. Likewise, detailed morphological characterization of both microfilariae and adult nematodes recovered from these hosts remains essential for accurately assessing filarioid diversity within Brazilian wildlife.

Beyond their taxonomic implications, the present findings also raise important ecological questions regarding the circulation of filarioids in central-western Brazil. Armadillos and anteaters have coexisted in this ecosystem for thousands of years, sharing habitats, foraging areas, and potentially exposure to the same assemblages of hematophagous arthropods that also feed on other wild and domestic vertebrates. Consequently, identifying the vectors involved in the transmission of these parasites represents a major knowledge gap. Very little is currently known about the hematophagous arthropods responsible for maintaining the transmission cycles of filarioids infecting xenarthrans. Therefore, elucidating these parasite–vector–host interactions will be essential for understanding the ecology, evolution, and maintenance of these filarioid nematodes in natural ecosystems.

Although the available necropsy records did not document any relevant gross findings or pathological alterations suggestive of filariosis in the road-killed animals that tested positive for filarioids in the present work, a previous study performed in Brazil reported ovarian filariosis in a road-killer adult female lesser anteater (*T. tetradactyla*) from Mato do Grosso do Sul state. The parasitized ovarian parenchyma presented adult nematodes (including females with microfilariae) surrounded by inflammatory infiltrates (Fromme et al. 2022). The real impact of ovarian filariosis on the reproductive success of wild southern tamanduas should be investigated in the future. Unfortunately, molecular confirmation followed by sequencing was not performed in the referred study, precluding additional inferences on the real identity of the involved filarioid specimens.

The results obtained in this study demonstrate a high rate of filarioid infection in armadillos and anteaters from the Pantanal and Cerrado biomes of Central-Western Brazil. Phylogenetic analyses based on distinct molecular markers (*cox1* and 12 S rRNA) revealed the circulation of genetically distinct filarioid lineages associated with mammals of the superorder Xenarthra in Brazil. This work represents the first molecular report of filarioids in armadillos.

## Data Availability

Sequences obtained in the present study were deposited in the NCBI Genbank (https://www.ncbi.nlm.nih.gov/genbank/) and can be accessed through the following accession numbers: PZ694553 (sample ES13), PZ694554 (sample ES31), PZ694555 (sample TC38), PZ694556 (sample ES12), PZ694557 (sample ES48), and PZ694558 (sample ES37) for the 12 S rRNA gene; and PZ782043 (sample NEC93), PZ782044 (sample NEC99), PZ782045 (sample ES12), PZ782046 (sample ES34), PZ782047 (sample ES48), and PZ782048 (sample ES43) for the *cox1* gene.
